# Wanted Badly

**Published:** 1866-05

**Authors:** 


### WANTED BADLY.

A good many sentimental tears have been shed over that phantasy of an inspirited brain, "The song of a shirt." There is no doubt the writer had a glass of brandy, and a pipe beside him, when he wrote all that rigmarole about "stitch, stitch, stitch." There is nothing like "leather," but hard facts. Some time ago, a friend wanted a dozen shirts made, and asked our aid in finding a seamstress; attracted by a sign, a young woman presented herself; she was asked to name her price for making one; and was told that if the work was well done she could have the remainder at her own price. The article was dilatorily made, sent home and paid for; the bosom buttons came off before the man was dressed; on examination, it was found that they were attached by a single thread, and even that, loosely. Who ever purchased a ready made garment that did not want repairing after a week's use, either as to buttons or rips. We feelingly know that a really good dressmaker commands two dollars a day, coming at eight o'clock and leaving at six, wanting two very hearty meals, with tea at both, two or three cups of it, of the strongest kind, costing one dollar and a half a pound; and to get this same woman, even for a day, is sometimes the work of a month, that is, she generally is engaged that long before hand; to be sure she understands her business, and does it well. A common sewer demands a dollar a day and board; but seldom can be had without a fortnight's notice; there are hundreds of families in New York city to day, who would gladly pay high prices for a person who could sew well. There are five thousand families in New York city who would cheerfully give from fifteen to twenty dollars a month for a cleanly, capable, honest and economical cook, who had no relations to feed; who had no visitors, and who retired at ten o'clock. There are five thousand places for house girls who are fully competent to the duties of their department. It is scarcely possible to go into any mechanical office or shop where work is well done, and get it promptly done, because such men have more work than they can do. It is only the incompetent who fail to do well in New York. Nor is it different in the professions; there is always a great demand for really able clergymen; men of might are wanted everywhere. There are a hundred churches in the city of New York ready and willing to pay large salaries to able men; the few who are here are constantly solicited to go elsewhere. As a good mechanic is never out of work; so a clergyman of real ability, is never out of a place long, if it is known that he is disengaged. We know a minister who is sixty years of age, who has never spent a Sabbath without a pastorate since he left the seminary; we know another who has pressing calls, with a princely salary, to New Orleans, St. Louis and San Francisco. Now and then a man of worth and power may stand awhile idle in the market place; bnt when it is so, it is because he is not appreciated, and is too retiring to push his claims. There are tens of thousands suffering this moment in the public hospitals, asylums and other places of charity, from destitution and diseases from want of occupation, not of necessity, but simply because they were either too idle to work, too incompetent to do it well, or too lazy to apply themselves.

We say to persons coming to the city to seek their fortunes, that they cannot get good places and high wages right away; but they can always be secured in a reasonable time, by accepting the first place offered, however small the salary, if it will provide very plain board and decent clothing; discharge all the duties promptly, well and cheerfully, and as your real merit becomes known, confidence will grow, salary will increase, and soon you will be considered indispensable, and in ten years become "one of the firm," and eventually, the head of the house on the retiring or death of your original employer. The most elegantly chaste house on Fifth avenue, within a stone's throw of our dwelling, is owned by a gentleman, who came to New York as a poor youth, and became "a store boy;" but he was economical, industrious and faithful, rising by degrees to clerk, confidential adviser, partner, and then principal, on the death of his employer. He is not now an old man, and his annual income would be considered a large fortune; but he never drank a glass of liquor, never smoked a cigar, never entered a theatre.

